# Chinese Herbal Medicines Attenuate Acute Pancreatitis: Pharmacological Activities and Mechanisms

**DOI:** 10.3389/fphar.2017.00216

**Published:** 2017-04-25

**Authors:** Hong Xiang, Qingkai Zhang, Bing Qi, Xufeng Tao, Shilin Xia, Huiyi Song, Jialin Qu, Dong Shang

**Affiliations:** ^1^College (Institute) of Integrative Medicine, Dalian Medical UniversityDalian, China; ^2^Department of General Surgery, The First Affiliated Hospital of Dalian Medical UniversityDalian, China; ^3^College of Pharmacy, Dalian Medical UniversityDalian, China; ^4^Clinical Laboratory of Integrative Medicine, The First Affiliated Hospital of Dalian Medical UniversityDalian, China

**Keywords:** acute pancreatitis, Chinese herbal formulas, natural products, pharmacological activities, toxic natural products

## Abstract

Acute pancreatitis (AP) is a commonly occurring gastrointestinal disorder. An increase in the annual incidence of AP has been observed, and it causes acute hospitalization and high mortality. The diagnosis and treatment guidelines for AP recommend conservative medical treatments focused on reducing pancreatic secretion and secondary injury, as a primary therapeutic approach. Unfortunately, the existing treatment options have limited impact on the incidence and severity of AP due to the complex and multifaceted pathological process of this disease. In recent decades, Chinese herbal medicines (CHMs) have been used as efficient therapeutic agents to attenuate AP in Asian countries. Despite early cell culture, animal models, and clinical trials, CHMs are capable of interacting with numerous molecular targets participating in the pathogenesis of AP; however, comprehensive, up-to-date communication in this field is not yet available. This review focuses on the pharmacological activities of CHMs against AP *in vitro* and *in vivo* and the underlying mechanisms. A computational prediction of few selected and promising plant-derived molecules (emodin, baicalin, resveratrol, curcumin, ligustrazine, and honokiol) to target numerous proteins or networks involved in AP was initially established based on a network pharmacology simulation. Moreover, we also summarized some potential toxic natural products for pancreas in order to more safe and reasonable medication. These breakthrough findings may have important implications for innovative drug research and the future development of treatments for AP.

## Introduction

**FIGURE 1:**
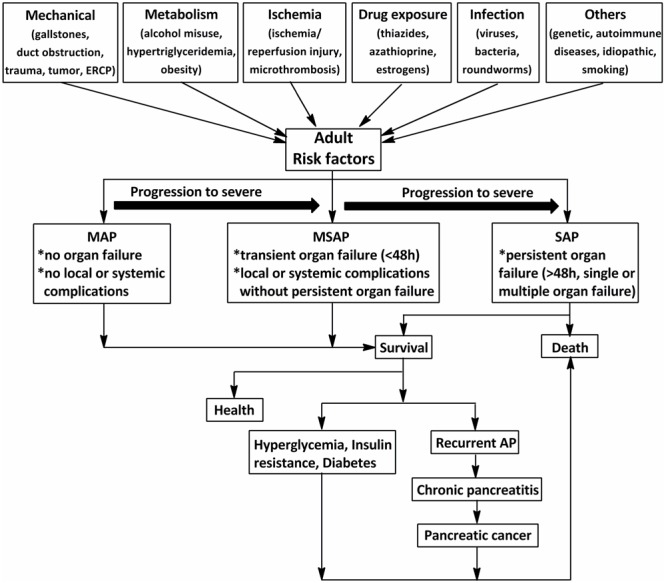
**The schematic of the procedure of acute pancreatitis (AP) in adults (>18 years).** The common risks for AP in adults are mechanical, metabolism, ischemia, drug exposure, infection, and so on. According to the 2012 revision of the Atlanta classification and definitions, AP should be divided into mild acute pancreatitis (MAP), moderately severe acute pancreatitis (MSAP), and severe acute pancreatitis (SAP). Although MAP and MSAP account for nearly 80% of AP cases, 20% of patients with SAP suffer persistent organ failure or even death. Patients with AP often develop prediabetes, diabetes and/or other complications after discharge from hospital. ERCP, endoscopic retrograde cholangiopancreatography.

Acute pancreatitis (AP) is characterized by a severe inflammatory response with the premature activation of pancreatic digestive enzymes, edema formation, cytoplasmic vacuolization, and infiltration of inflammatory cells into the pancreas (Yadav and Lowenfels, 2006; Banks et al., 2013). The most common risk for AP in adults is gallstones, which increases with age (Lowenfels et al., 2000). Excessive alcohol consumption, as the second-most common cause of AP after gallstones, has been shown to increase the risk of pancreatitis in a dose-dependent manner (Lankisch et al., 2002, 2015). The incidence of AP among subjects with long-term alcoholism is fourfold higher than those without (Yadav et al., 2007). Other causes include duct obstruction (e.g., related to a tumor or anatomic abnormalities), metabolic aberrations (e.g., hypertriglyceridemia), drug exposure (e.g., thiazides, azathioprine, and estrogens), smoking, and trauma (Yadav and Lowenfels, 2013; Lankisch et al., 2015) (**Figure 1**). Systemic diseases and trauma are particularly common in pediatric patients with AP and differ from those in adults (Snyder, 2000).

Acute pancreatitis is one of the most frequent gastrointestinal diseases leading to total hospital stays, with a reported global annual incidence of 13–45 per 100 000 people (Lankisch et al., 2015). The number of discharges with AP as the principal diagnosis in the USA in 2009 increased 30% compared with that in the year 2000 (Lankisch et al., 2015). Although nearly 80% of patients with AP exhibit mild symptoms and are easy to treat, 20% of those suffer a severe attack with progression to systemic inflammatory response syndrome (SIRS) and multiple organ dysfunction syndromes (MODS), and approximately 10–30% of patients with severe acute pancreatitis (SAP) may die (Whitcomb, 2006). In addition, as shown in **Figure 1**, the associations between AP and certain chronic diseases have garnered attention in AP research in the past 10 years (Yadav et al., 2012; Gillies et al., 2016; Kennedy et al., 2016). Investigations from the US showed a transition to chronic and pancreatic cancer from AP in 32.3% after 3.4 years (Yadav et al., 2012). Findings from a cross-sectional follow-up study and a recent comprehensive systematic review indicate that approximately 40% of patients may develop newly diagnosed prediabetes (e.g., chronic hyperglycemia, insulin resistance) or diabetes after AP, with the risk of diabetes doubling within 5 years (Gillies et al., 2016; Kennedy et al., 2016). Therefore, approaches with high efficacy and minimal side effects are imperative for the treatment of AP.

Since the first international classification of pancreatitis formulated during the 1963 Marseille meeting, the management guidelines of AP have been enacted one after another; so far, a preliminary consensus regarding the treatment for AP has been established (Tenner et al., 2013; Working Group IAP/APA Acute Pancreatitis Guidelines, 2013; Yokoe et al., 2015). Except for a few acute hemorrhagic necrotizing pancreatitis (AHNP) cases that require surgery, the primary therapeutic approach is to recommend conservative treatment focused on reducing pancreatic secretion and secondary injury, including fasting, fluid resuscitation, protease inhibitors, and antibiotics (Whitcomb, 2006; Banks et al., 2013; Lankisch et al., 2015). Although these strategies have been verified in randomized controlled trials, they have a limited impact on the incidence and severity of AP due to their unpredictable side effects and poor patient compliance. So far, AP is still a significant and unresolved challenge to clinicians. Hence, there is a huge demand to explore novel candidates for AP treatment.

Chinese herbal medicines (CHMs), which are abundant sources of biologically active substances, have been commonly used in clinical practices in many countries (Normile, 2003). Presently, more and more CHMs (including Chinese herbal formulas and pleiotropic natural products) have been discovered to have potent effects against AP through targeting numerous protein or biological networks involved in this disease (Tian et al., 2009; Chen et al., 2011; Xiang et al., 2016). Thus, CHMs are promising candidate drugs for the treatment of AP compared with western medicine, which usually focuses on a single target. For the future development of innovative medicines and to extend the influence of CHMs worldwide, we systematically summarize the available Chinese herbal formulas and pleiotropic natural products used in the treatment of AP and discuss their underlying mechanisms in this review. Within this frame, the underlying mechanisms of AP will also be covered.

## Underlying Mechanisms of AP

**FIGURE 2:**
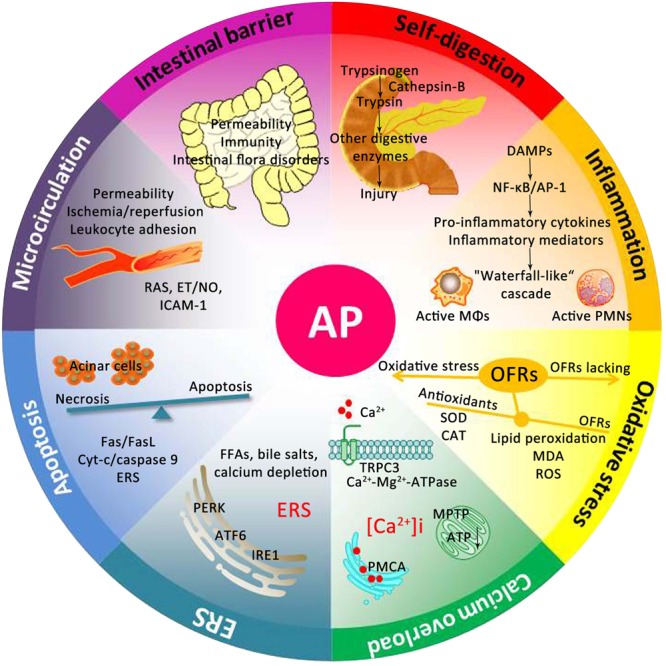
**Underlying mechanisms of the occurrence and development of AP.** DAMPs, damage associated molecular patterns; NF-κB, nuclear factor kappa-B; AP-1, activator protein-1; MΦs, macrophages; PMNs, polymorphonuclear neutrophils; OFRs, oxygen free radicals; SOD, superoxide dismutase; CAT, catalase; MDA, malondialdehyde; ROS, reactive oxygen species; TRPC3, transient receptor potential channel 3; MPTP, mitochondrial permeability transition pore; ATP, adenosine triphosphate; PMCA, plasma membrane calcium ATPase; FFAs, free fatty acids; ERS, endoplasmic reticulum stress; PERK, RNA-activated protein kinase-like ER kinase; ATF6, activation transcription factor 6; IRE1, inositol requiring protein 1; Cyt-c, Cytochrome *c*; RAS, renin-angiotensin system; ET, endothelin; NO, nitric oxide; ICAM-1, intercellular adhesion molecule-1.

For centuries, many theories have been proposed to explain the underlying mechanism of AP. As presented in **Figure 2**, the pathogenesis of AP seems to be related to a series of complex and multifaceted pathological processes, involving pancreatic self-digestion (Lerch and Gorelick, 2000), inflammatory response, oxidative stress (Xiang et al., 2016), intracellular calcium overload, endoplasmic reticulum (ER) stress (Wu J.S. et al., 2016), pancreatic acinar cell apoptosis and necrosis (Xiang et al., 2016), and microcirculation disorder (Tomkotter et al., 2016). Current therapies, which target the above pathological mechanisms, may improve the prognosis of AP.

### Pancreatic Self-digestion Theory

The “pancreatic self-digestion theory” was proposed by Chiara et al. (1886) for the first time (Jin et al., 2013), and this theory suggested that pancreatic duct obstruction and the blockage of pancreatic juice outflow under pathological conditions are associated with exocrine pancreatic hyperstimulation and active trypsin reflux and result in the autodigestion of the pancreas and pancreatitis (Lerch and Gorelick, 2000). This theory was supported by research executed in cerulean-induced AP mice with or without the trypsinogen isoform 7 (T7) gene (T^-^/^-^). Unlike zymogen activation and AP in wild-type mice, necrosis and cell death were significantly reduced in T^-^/^-^ mice (Dawra et al., 2011). Most researchers accept that the direct trigger for the onset and exacerbation of AP relates to the inappropriate activation of trypsinogen to trypsin, the key enzyme in the activation of additional zymogens, and a lack of prompt elimination of active zymogens (Lerch and Gorelick, 2000; Whitcomb, 2006). Pancreatic acinar cells are specialized for the production, storage, and release of pancreatic zymogens (Hofbauer et al., 1998). During AP, lysosomal enzymes are mistargeted to the organelles containing zymogens within the acinar cell. The lysosomal hydrolase cathepsin-B prematurely activates trypsinogen, whereas the inhibition or knockout of cathepsin-B has been shown to relieve trypsinogen activation and acinar cell damage (Saluja et al., 1997; Van Acker et al., 2002).

In addition, there are a variety of protective mechanisms against premature zymogen activation *in vivo* through the inhibition or degradation of activated trypsin (Hirota et al., 2006; Binker et al., 2015). These mechanisms include inhibition by pancreatic secretory trypsin inhibitor (PSTI), also known as a serine protease inhibitor Kazal-type 1 (SPINK1) or tumor-associated trypsin inhibitor (TATI) (Hirota et al., 2006), and degradation by chymotrypsin-C (CTRC) (Binker et al., 2015) and the lysosomal hydrolase cathepsin-L (Wartmann et al., 2010). Once these protective mechanisms are expended, there is an increased risk of developing AP.

### Inflammatory Response

An acute inflammatory cascade is shown to be the main reason that mild acute pancreatitis progresses to SIRS and MODS in response to pancreatic cell injury (Whitcomb, 2006; Lankisch et al., 2015). In recent years, studies suggested that damage associated molecular patterns (DAMPs), mainly high mobility group box 1 (HMGB1) protein, are released by injured and necrotic acinar cells (Scaffidi et al., 2002). HMGB1 is an intracellular DNA-binding protein involved in neutrophil activation and pro-inflammatory factor secretion via Toll-like receptors (TLRs) in the pathogenesis of AP (Shen and Li, 2015). TLR4 is the first target response to extracellular HMGB1 and can activate the myeloid differentiation primary response gene 88 (Myd88)-dependent pathway, TNF-associated factor 6 and the MAPK signal transduction pathway, which then lead to the activation of nuclear factor kappa-B (NF-κB) and activator protein-1 (AP-1) (Li et al., 2016b). NF-κB and AP-1 are known transcription factors with multiple functions, required for the early regulation of inflammatory signaling (Yang et al., 2016). NF-κB in the cell cytoplasm is bound to the inhibitor protein kappa B (IκB) in an inactive form. During cellular stress, IκB is phosphorylated by specific IκB kinase (IKK) and rapidly degraded via the proteasome-dependent pathways (Hayden and Ghosh, 2008). NF-kB activation during AP is capable of up-regulating the expression of cytokines, chemokines and adhesion molecules, the activation of macrophages, and the infiltration of neutrophils and lymphocytes into the pancreas and peritoneum, thus amplifying the inflammatory response (Jakkampudi et al., 2016). AP-1 is a heterodimer complex formed from proteins belonging to the c-Fos, c-Jun, and activating transcription factor (ATF) families (Schraml et al., 2009). AP-1 can be activated by multiple factors such as growth factors, cytokines, and chemokines, which often act in concert with NF-κB to control the cascade chain reaction of inflammatory mediators (Koh et al., 2010). The mechanism of AP-1 activation involves the phosphorylation of c-Jun (Schraml et al., 2009).

During AP, neutrophils migrate from the bloodstream to the damaged tissues, significantly increasing the numbers of local neutrophils in the pancreas (Yang Z.W. et al., 2015). Activated neutrophils have the capability to exacerbate the release of many types of pro-inflammatory cytokines and inflammatory mediators, which contain tumor necrosis factor-α (TNF-α), interleukin (IL)-1, IL-6, and IL-8, oxygen free radicals (OFRs), platelet-activating factor (PAF), leukotrienes, and thromboxane A2 (TXA2), and many enzymes, in particular, elastase and phospholipase A2 (PLA2) (Kolaczkowska and Kubes, 2013; Nauseef and Borregaard, 2014; Yang Z.W. et al., 2015). These released signaling molecules, in turn, activate NF-kB via highly specific receptor binding patterns, leading to pancreatic inflammation, necrosis and microcirculation disorder, and the release of excess endotoxin (Rotstein, 2014). The inflammatory response is constantly activated, not only in the local pancreas but also in the extrapancreas, and evolves into multiple organ injury and SIRS.

### Oxidative Stress

Kishimoto et al. (1995) found that pancreatic OFRs increased after the induction of AP in rats, demonstrating that significant peroxidation occurs in AP. In patients with AP, the involvement of OFRs is independent of the underlying etiology (Park et al., 2003). Upon physiological conditions, the production and elimination of OFRs remain balanced. OFRs accumulate in the pancreas during AP development, which can attack macromolecules, such as proteins, lipids, and polysaccharides inside the biomembrane, initiating lipid peroxidation and resulting in the breakage of membrane stability and the release of zymogen granules in the acinar cell (Chvanov et al., 2005; Perez et al., 2015). OFRs activate PLA that can break down lecithinum inside the cellular membrane and further initiate pancreatic edema, hemorrhage, degeneration, and necrosis (Tsukahara et al., 1999). Moreover, many OFRs released in AP also cause NF-κB and AP-1 activation, antioxidant consumption, and superoxide dismutase (SOD) activity decrease, which in turn causes lipid peroxidation and pancreas damage (Yu and Kim, 2014; Perez et al., 2015).

### Calcium Overload

In general, calcium release, uptake and extrusion mechanisms remain in fine coordination, whereas some factors can influence calcium regulation and increase the intracellular calcium concentration under certain conditions (Criddle, 2016). Cytosolic calcium is a secondary messenger of intracellular signal transduction and applies to many cellular events, including the regulation of trypsin activity (Kruger et al., 2000). In AP, calcium homeostasis is disrupted, resulting in calcium overload within a cell, which can aggravate the disease state (Kruger et al., 2000; Maleth and Hegyi, 2014). Studies have shown that hypercalcemia causes histological damage during AP in a time- and dose-dependent manner (Gerasimenko et al., 2014). Calcium overload within the pancreatic acinar cells can induce mitochondrial impairment through the formation of the mitochondrial permeability transition pore (MPTP) and the depletion of ATP and necrosis, thus leading to serious injury of the acinar cells and pancreatic inflammation (Mukherjee et al., 2016).

The maintenance of intracellular calcium homeostasis depends on a stable mechanism that coordinates calcium release, entry and exit (Criddle, 2016). It is known that calcium release from internal stores occurs via inositol 1,4,5-trisphosphate receptor (IP3R)-dependent calcium oscillations that promote exocrine secretion (Camello-Almaraz et al., 2006). There are several ion channels (TRPC3 channels, STIM1-Orai complex) in pancreatic acinar cells implicated in important calcium entry mechanisms, which mediate sustained pathophysiological elevations in intracellular calcium causing mitochondrial injury and cell death (Kim et al., 2009; Lur et al., 2009). Considering the benefit of the production and supply of intracellular adenosine triphosphate (ATP) for intracellular calcium homeostasis in the pancreatic acinar cell, recent studies reported that calcium clearance requires ATP-dependent pumps modulated by sustained oxidative stress (Bruce and Elliott, 2007). In AP, the structural integrity of the plasma membrane is destroyed, and the quantity and activity of endocytoplasmic/sarcoplasmic reticulum (SR) membrane Ca^2+^-ATPase (PMCA) and cytomembrane Ca^2+^-Mg^2+^-ATPase are decreased, and the intracellular calcium is not pumped out of the cell or back into the calcium stores in time (Baggaley et al., 2008; Wang et al., 2008).

### ER Stress

The acinar cells of the exocrine pancreas have an abundant ER that contributes to the synthesis and secretion of proteins. ER enzymes require optimal ion concentrations (e.g., calcium) and redox conditions for their regulation and function (Kubisch and Logsdon, 2008). Excess free fatty acids (FFAs), bile salts, calcium depletion of the ER stores, and oxidative stress activate the ER stress response and the injury of acinar cells, which initiates a complicated cascade of events during the early stage of AP (Zeng et al., 2012; Wu et al., 2013). RNA-activated protein kinase-like ER kinase (PERK), activation transcription factor 6 (ATF6), and inositol requiring protein 1 (IRE1) are believed to be ER stress sensors, which play a key role in transducing the stress signals from the ER to the cytoplasm (Kubisch and Logsdon, 2008). In AP, these signaling molecules and their downstream molecules TNF receptor-associated factor 2 (TRAF2) and apoptosis signal-regulating kinase 1 (ASK1) are highly activated (Sendler et al., 2013). This activation parallels trypsinogen activation and leads to apoptosis and inflammatory and immune responses (Kubisch and Logsdon, 2007).

### Cell Apoptosis

Both necrosis and apoptosis are the two types of crucial cell death in AP, and may interchange under appropriate conditions. Recent studies prove that the regulation of the necrosis/apoptosis switch is beneficial for alleviating the severity of AP because apoptotic cells maintain membrane integrity without triggering an inflammatory cascade, unlike necrotic cells, which release intracellular contents containing pro-inflammatory and immunogenic cytokines (Takeyama, 2005; Fu et al., 2016). Apoptosis is controlled via three known pathways: the death receptor, the mitochondrial pathway, and the ER stress-induced apoptotic pathway. In early SAP, the stimulation of death receptors such as Fas and tumor necrosis factor receptor 1 (TNFR1) leads to the formation of a death-inducing signaling complex and initiator caspase-8, which initiates downstream caspase-3 (Zhang et al., 2007a). Death receptors transmit the death signals to mitochondria resulting in the release of cytochrome *c* (a precursor of apoptosis) into the cytoplasm through a permeability transition pore in the mitochondrial membrane, which subsequently binds to the apoptotic protease activating factor-1 (Apaf-1) and procaspase-9 to form the apoptosome and activate caspase-3 (Xia et al., 2002). Bcl-2, an anti-apoptotic member of the Bcl-2 family, can break the opening of the permeability transition pore induced by Bax (Ma et al., 2011). Recent studies suggest several molecules such as IRE1, C/EBP homologous protein (CHOP), Bcl-2 family and caspase-12 play a role in ER stress-induced apoptosis (Morishima et al., 2002). Evidence indicates that the sustained activation of IRE1 enhances cell survival, suggesting a link between ER stress and death cell fate. CHOP transcription is induced by the activation of PERK and ATF6. Apoptosis via the CHOP signaling pathways is mitochondria-dependent because CHOP can break down the membrane potential and promote the release of cytochrome *c* from mitochondria (Kubisch and Logsdon, 2008). The members of the ER-resident proapoptotic Bcl-2 family, Bak and Bax, can maintain the homeostasis of ER calcium. When ER stress and increased cytosolic calcium correlate with an increase in calpain activation, it contributes to the activation of ER-resident caspase-12, which directly excites caspase-3 and leads to cellular apoptosis (Nakagawa and Yuan, 2000). Bcl-2 can clear OFRs and prevent the release of calcium from ER and leads to necrosis by inhibiting apoptosis (Wu and Tang, 2016).

### Microcirculation Disorder

Microcirculation obstruction throughout the development of AP is a systemic response to pancreatic injury and is closely associated with MODS (Zhou and Chen, 2002; Ma et al., 2011; Tomkotter et al., 2016). The pancreas is the main site of the abnormal metabolism of eicosanoids (TXA2 and PGI2), which are decomposed into several types of inflammatory mediators, such as PAF and leukotrienes, thereby aggravating the inflammatory reaction and microcirculatory disturbance (Zhang et al., 2009b). Microcirculatory hypoperfusion causes the inflow of calcium into cells, leading to calcium overload in the pancreatic cells (Zhou et al., 2004). Calcium influx activates the phospholipid cell system, leading to the destruction of the lysosomal membrane and the release of the enzyme and large amounts of toxic media (Maleth and Hegyi, 2014). In addition, significant hemorrhagic changes in a rat model of SAP have been reported, including high hematocrit (HCT) and blood viscosity, as well as microthrombus formation. The possible mechanism relates to the activation of pancreatin that increases the permeability of capillary vessels, resulting in the exudation of lots of plasma-like liquids from the blood circulation to tissue space. Fragmented erythrocytes release tissue factors, such as adenosine diphosphate (ADP), which can activate the coagulation system and consume abundant fibrinogen (Ma et al., 2011). These factors may be the primary causes of progressive pancreatic tissue necrosis and MODS.

### Altered Gut Barrier Permeability

The gut mucosal barrier serves as a “natural guard” in the human body, preventing the entrance of potentially harmful intestinal bacteria and endotoxin into the systemic circulation and extra-intestinal tissues. The gut acts as an important target organ in response to SAP, and as the main source of pancreatic bacterial super infection and related septic complications (Wang et al., 2016). Gram-negative enteric-type organisms are shown to be the culprit in most pancreatic and peripancreatic infections (Hanna et al., 2014). In SAP early, hypovolemia, splanchnic vasoconstriction, and ischemia-reperfusion injury initiates a derangement in gut barrier function. Increased intestinal permeability, physical and chemical factor changes, and cell immune dysfunction induced by impaired gut mucosa barrier destroy normal flora structure and promote intestinal lumen-derived bacteria and endotoxins translocation to other organs through blood circulation or the lymphatic system, or through a pancreatic duct or bile duct directly connected with the intestinal tract (Guo et al., 2014). Intestinal microflora can also directly penetrate the damaged intestinal mucosal barrier into the abdominal cavity. In this scenario, an augmented immune response is triggered, which causes the gut to become a pro-inflammatory organ that releases cytokines, chemokines, and other pro-inflammatory intermediates. These mediators cause a “waterfall-like” inflammation cascade via the excessive activation of macrophages and neutrophils and release of oxidant and proteolytic enzymes, which aggravates secondary infection in the pancreas, eventually resulting in SIRS and MODS (Leveau et al., 1996; Wu et al., 1998).

## Chinese Herbal Formulas

Chinese herbal formulas have upheld the holistic therapeutic philosophy for thousands of years, which consists of two or more appropriate medicinal plants or animals according to the prescription compatibility principle of traditional Chinese medicine (TCM) formulations and determining the dosage and usage of each medicine (Normile, 2003; Yao et al., 2016b). The components of Chinese herbal formulas are complex and diverse, and the main treatment mechanism seems reasonable. These herbal formulas are an organic combination of many effective components having a multi-target effect on the disease in the body by multiple pathways (Cheng et al., 2015; Wu T.Y. et al., 2016; Xiang et al., 2016). An increasing number of Chinese herbal formulas have been reported to have significant anti-AP effects, and have become a treatment option in many hospitals for AP (**Table 1**).

**Table 1:** List of traditional Chinese medicine formulas for the treatment of AP.

Formula	Common composition	Mechanisms	Reference
DCQD	*Radix Rhei* Et Rhizome (Dahuang), *Magnolia Officinalis Rehd* Et Wils (Houpu), Aurantii Fructus Immaturus (Zhishi), Natrii Sulphas (Mangxiao)	↓CRP, IL-6, TNF-α, L/M ratio, LPS↓ROS, ↑NO, iNOS↓HMGB1, TLRs, NF-κB, p38 MAPK, IL-6, TNF-α	[Bibr B10][Bibr B125][Bibr B13]

DCQD (Modified)	*Radix Rhei* Et Rhizome (Dahuang), Thenardite (Xuanmingfen), *Magnolia Officinalis Rehd* Et Wils (Houpu), Aurantii Fructus (Zhiqiao), Persicae Semen (Taoren), Raphani Semen (Laifuzi)	↓HMGB1, TNF-α	Qin et al., 2015

CQCQD	Radix Bupleuri (Chaihu), Scutellariae Radix (Huangqin), *Magnolia Officinalis Rehd* Et Wils (Houpu), Aurantii Fructus Immaturus (Zhishi), Artemisiae Scopariae Herba (Yinchen), Fructus Gardeniae (Zhizi), *Radix Rhei* Et Rhizome (Dahuang), Natrii Sulfas (Mangxiao)	↑SERCA2↓NF-κB, TNF-α, IL6↓[Ca^2+^]i↓CCKR1, PLC, IP3↑Cytochrome *c*, Caspase-3↑nAChRalpha7, Ach, ↓IL6	[Bibr B154][Bibr B71][Bibr B20][Bibr B34][Bibr B73][Bibr B155]

CQCQD (Modified)	Radix Bupleuri (Chaihu), Scutellariae Radix (Huangqin), *Magnolia Officinalis Rehd* Et Wils (Houpu), Aurantii Fructus Immaturus (Zhishi), *Radix Rhei* Et Rhizome (Dahuang), Cortex Moutan (Danpi), Corydalis Rhizoma (Yuanhu), Toosendan Fructus (Chuanlian), Kansui Radix (Gansui), Natrii Sulphas (Mangxiao)	↓SAA↓ MMP-9	Wu et al., 2012;Guo et al., 2013

QYD	Gardenia jasminoides Ellis (Zhizi), Cortex Moutan (Danpi), Radix Paeoniae Rubra (Chishao), Aucklandiae Radix (Muxiang), *Magnolia Officinalis Rehd* Et Wils (Houpu), Corydalis Rhizoma (Yuanhu), *Radix Rhei* Et Rhizome (Dahuang), Natrii Sulphas (Mangxiao)	↓Genes: Rgs2, Pnlip, Cpa2, Ela2, LOC503278, Sv2b, LOC500909, Cln3, Reg1, Fbxl20↑Genes: Glrx1, LOC499457, Txnl2, Eef1g, LOC499793, Rpl10, LOC499906, Dap, Eef1b2, LOC362290	Zhu et al., 2014

QYD (Modified)	*Radix Rhei* Et Rhizome (Dahuang), Natrii Sulphas (Mangxiao), Kansui Radix (Gansui), Radix Bupleuri (Chaihu), Aucklandiae Radix (Muxiang), Scutellariae Radix (Huangqin), Coptidis Rhizoma (Huanglian), *Magnolia Officinalis Rehd* Et Wils (Houpu), Gardenia jasminoides Ellis (Zhizi), Paeoniae Radix Alba (Baishao), Persicae Semen (Taoren)	↓NF-κB, TNF-α, IL-6, IL-8	Yang et al., 2009

QYG	*Radix Rhei* Et Rhizome (Dahuang), Radix Bupleuri (Chaihu), Hedysarum Multijugum Maxim (Huangqi), Natrii Sulfas (Mangxiao), Paeoniae Radix Alba (Baishao), Aucklandiae Radix (Muxiang)	↓Proteins: Serpinbla 43 KDa, ClpS, Actg1, Eprs, Hadhsc↑Proteins: Serpinbla 39 KDa, Prx-IV	Yang et al., 2013

YCHD	Artemisia capillaris Thunb. (Yinchen), Gardenia jasminoides Ellis (Zhizi), Rheum officinale Baill. (Dahuang)	↑PPARγ, ↓NF-κB	Xiang et al., 2016

LHD	*Radix Rhei* Et Rhizome (Dahuang), Cortex Phellodendri Chinensis (Huangbai), Rhizoma Bletillae (Baiji), Radix Angelicae Dahuricae (Baizhi), Fructus Mume (Wumei), Herba Menthae (Bohe)	↓TNF-α, IL-6, IL-10, ↑SOD	Peng et al., 2013

TXHYD	*Radix Rhei* Et Rhizome (Dahuang), Natrii Sulfas (Mangxiao), Aurantii Fructus Immaturus (Zhishi), Curcumae Radix (Yujin), *Polygoni Cuspidati Rhizoma* Et Radix (Huzhang), Cortex Moutan (Mudanpi), Radix Paeoniae Rubra (Chishao), Corydalis Rhizoma (Yanhusuo)	↓ET	Fang et al., 2007

YHQYD	*Radix Rhei* Et Rhizome (Dahuang), Natrii Sulfas (Mangxiao), Scutellariae Radix (Huangqin), Paeoniae Radix Alba (Baishao), Radix Ophiopogonis (Maidong), Aucklandiae Radix (Muxiang),	↑Pancreatic blood flow	Chen et al., 2011

	Radix Bupleuri (Chaihu), Semen Arecae (Binglang), Cortex Meliae (Kulianpi), Fructus Quisqualis (Shijunzi), Rhizoma Chuanxiong (Chuanxiong), Salviae Miltiorrhizae (Danshen), Radix Astragali (Huangqi), Radix Ginseng (Renshen)		

CHSHD	Radix Bupleuri (Chaihu), *Radix Rhei* Et Rhizome (Dahuang), Scutellariae Radix (Huangqin), Coptidis Rhizoma (Huanglian), Phellodendri Chinensis Cortex (Huangbai), Radix Paeoniae Rubra (Chishao), Radix Salviae (Danshen)	↓TNF-α, IL-6, ↑IL10	Wang H. et al., 2009

*DCQD, Dachengqi decoction; CQCQD, Chaiqinchengqi decoction; QYD, Qingyi decoction; QYG, Qingyi granule; YCHD, Yinchenhao decoction; LHD, Liuhedan; TXHYD, Tongxiahuayu decoction; YHQYD, Yihuoqingyi decoction; CHSHD, Chaihusihuang decoction; CRP, C-reactive protein; IL, interleukin; TNF-α, tumor necrosis factor-α; L/M, lactulose/mannitol; LPS, lipopolysaccharide; ROS, reactive oxygen species; NO, nitric oxide; iNOS, inducible nitric oxide synthase; HMGB1, high mobility group box 1; TLRs, Toll-like receptors; NF-κB, nuclear factor kappa-B; p38 MAPK, p38 mitogen-activated protein kinases; SAA, serum amyloid A; MMP-9, matrix metalloproteinase 9; SERCA2, sarco/endoplasmic reticulum Ca^*2+*^-ATPase; CCKR1, cholecystokinin receptor 1; PLC, phospholipase C; IP3, inositol-1,4,5-triphosphate; nAChRalpha7, neuronal acetylcholine receptor alpha 7; Ach, acetylcholine; PPARγ, peroxisome proliferator-activated receptor gamma; SOD, superoxide dismutase; ET, endothelin.*

Dachengqi decoction (DCQD), first documented in “*Shang Han Lun”* (Treatise on Febrile Diseases), is a representative purgative for constipation treatment and for clearing internal heat in the gastrointestinal tract. DCQD consists of *Radix et Rhizoma Rhei, Cortex Magnoliae Officinalis, Fructus Aurantii Immaturus*, and *Natrii Sulphas*, which have been used as a classical prescription in China to treat AP for more than three decades (Chen et al., 2010). Rhein, naringin, and honokiol may be the major effect components of DCQD in treatment of AP (Zhang et al., 2016). Clinical studies indicated that DCQD could decrease the ratio of lactulose (L)/mannitol (M) and the surrogate of intestinal permeability assays, which suggested that DCQD protect the intestinal mucosal immune barrier and decrease the incidence of pancreatic infection and MODS (Chen et al., 2010; Jiang, 2010).

On the molecular mechanism, DCQD decreases pro-inflammatory cytokines and alleviates the severity of AP through inhibiting TLR/HMGB-1 signal pathways (Chen et al., 2010, 2015; Qin et al., 2015). DCQD could also render pancreas more resilient to stress and microcirculation disorder through eliminating excessive reactive oxygen species (ROS), inducing apoptosis and relieving the necrosis in acinar cells (Ren et al., 2009; Wang et al., 2012). Moreover, recent studies have shown that DCQD is an effective digestive kinetic agent that could promote the gastrointestinal motility and the recovery of intestinal mucosal permeability, and affect the bacterial translocation in the animal models of AP (Chen et al., 2010; Qin et al., 2015).

Chaiqinchengqi decoction (CQCQD), modified from DCQD, is a traditional Chinese prescription used as a purgative. In recent decades, CQCQD has shown significant efficacy in the treatment of AP both *in vivo* and *in vitro* (Liu et al., 2004; Deng et al., 2008a; Xue et al., 2008; Wang et al., 2011). Clinical trials have also proven that CQCQD significantly relieves the severity of clinical symptoms, reduces the duration of organ damage, and shortens the hospitalization time of AP patients (Wang et al., 2011). Research showed that CQCQD may have efficient actions on the activation of choline acetyl transferase (ChAT) and neuronal acetylcholine receptor alpha 7 (nAChRα7) in peritoneal macrophages, which could inhibit the release of active macrophage pro-inflammatory cytokines (Li et al., 2008; Wang et al., 2011; Xue et al., 2014; Wu W.U. et al., 2016). CQCQD also inhibit the exocrine function of the pancreatic acinar cells and relieve pancreatic tissue lesions via reducing the overload of intracellular calcium (Deng et al., 2008a,b; Xue et al., 2008; Guo et al., 2015). In addition, CQCQD also regulates necrosis to apoptosis in pancreatic cells by promoting the release of mitochondrial cytochrome *c* and increasing pancreatic caspase-3 activity in SAP rats (Lin et al., 2014). Moreover, CQCQD has a protective effect in SAP complicated with acute lung injury (ALI) and acute respiratory distress syndrome by inhibiting ER stress in AMs, attenuating pro-inflammatory cytokine release and paracellular leakage, which involved in the down-regulation of p-Src, p-p85α, and c-Fos (Wang Z.C. et al., 2009; Jia et al., 2015; Wu W.U. et al., 2016). In addition, the modified CQCQD can relieve the severity of clinical symptoms in patients with SAP via lowering serum amyloid A (SAA) and matrix metalloproteinase 9 (MMP-9) (Wu et al., 2012; Guo et al., 2013).

Another common Chinese herbal decoction, qingyi decoction (QYD) or qingyi granule (QYG) is generally well tolerated by patients and exhibits purgative function, eliminates blood stasis, promotes blood circulation, and reduces inflammation in the pathogenesis of AP (Yang et al., 2009, 2013; Zhu et al., 2014). Moreover, clinical research proved that QYD could ameliorate AP-induced intestinal barrier injury by inhibiting the expression of intestinal secreted phospholipase A2 (sPLA2) (Zhang et al., 2015). Furthermore, an Illumina whole genome expression profile analysis for pancreatic RNA expression of SAP rats screens 575 differential genes between the SAP and QYD group, including 92 up-regulated genes and 483 down-regulated genes; and the Gene Ontology (GO) categories indicated that these genes are involved the MAPK and NLR signaling pathways, metabolic pathways, cell cycle, and oxide reductase activities (Yang et al., 2013; Zhu et al., 2014). In addition, QYD can reduce the extent of extrapancreatic organ injuries in SAP. QYD administration not only reduces SAP-induced ALI by reducing alveolar type II epithelial cell (AEC II) apoptosis, inhibiting the overexpression of secretory type II phospholipase A2 (sPLA2) (Liu et al., 2014), but also improves SAP complicated liver and renal injuries through decreasing HMGB1 expression (Yang Y.S. et al., 2015). In addition, the actions of modified QYD involve in the biological processes include directly neutralizing endotoxins, reducing intestinal endotoxin generation and absorption, inhibiting excessive neutrophil activation and NF-κB expression, and minimizing the release of inflammatory cytokines (Yang et al., 2009).

Moreover, our previous study found that Yinchenhao decoction (YCHD), known as an anti-inflammatory and choleretic agent, may also be a potential therapy for AP through pro-apoptosis, anti-inflammation, anti-oxidation, and regulation of lipid metabolism partially via regulating the NF-kB/PPARγ signaling pathway (Xiang et al., 2016). Apart from these, other Chinese herbal formulas including Liuhedan (Peng et al., 2013), Huoxueingyidecoction (Ji et al., 2016), Tongxiahuayu decoction (Fang et al., 2007), Yihuoqingyi decoction (Chen et al., 2011), Chaihusihuang decoction (Wang H. et al., 2009), Dachaihu decoction (Cheng et al., 2008), and Qingyichengqi decoction (Zhang et al., 2014) are also effective treatments for AP.

Despite the promising effects of Chinese herbal formulas, huge differences between TCM and modern medicine still push TCM away from mainstream medicine (Liu et al., 2016). There are several issues and challenges in current studies: First, the drawbacks of traditional decoctions and its administration are obvious, such as discommodiousness, unstable efficacy, and uncontrollable quality. Second, the data that harvested from rigorously stochastic, double-blind, placebo-controlled trials with multiple centers and large samples is insufficient (Qiong et al., 2005). Third, the interaction between the complex chemical and biological systems of AP remains ambiguous. Finally, the pharmacokinetics of these formulas and their interactions with other medications should be further evaluated (Yao et al., 2016b).

## Pleiotropic Natural Products

Pleiotropic natural products derived from medicinal plants through extraction, separation, and purification have clear chemical structures that are different from Chinese herbal formulas and crude extracts (de Oliveira et al., 2015; Bulaj et al., 2016; Yao et al., 2016b). Pure natural products, as carriers of modern herbal medicine advanced technology, are considered as the only way for TCM to realize modernization and internationalization (Normile, 2003; Yao et al., 2016b). Natural products have been used in Japan since the 1970s and have been widely developed in Singapore and other places (Normile, 2003). In Europe and the United States, 25% of prescriptions contain at least one extract from higher plants or compounds. In Germany, pure natural products are considered as drugs rather than food supplements and are covered by medical insurance (Yao et al., 2016a). In short, pure natural products in foreign countries have a good application base and a wide range of markets.

Pleiotropic natural products feature the following advantages: (1) well-known molecular weight, physical and chemical characteristics and easy qualitative and quantitative analyses; (2) various technique indexes can be controlled in the preparation process, and strict quality control can be respected; (3) the concentration of TCM effective ingredients can be greatly increased, enhancing medicine absorption in the body, thereby overcoming the fatal weakness of the slow effect of TCM; and (4) the pollution of harmful substances such as heavy metal ions and residual pesticide, can be maximally eliminated (Zhang et al., 2008b, 2009). Many pure natural products such as emodin, baicalin, resveratrol, and curcumin have been found to have significant therapeutic benefits against AP (**Table 2**). However, there is lack of clinical researches about the doses and side effects of these natural products at present.

**Table 2:** List of pure natural products derived from medicinal plants for the treatment of AP.

Natural products	Main source	Models	Mechanisms	Reference
Emodin	*Rheum palmatum* L., *Polygonum cuspidatum, Polygonum multiflorum, Aloe vera, Cassia obtusifolia, Radix et Rhizoma Rhei*	SAP rats (NaTc)AP rats (NaTc)SAP rats (NaTc)AR42J cells (caerulein + LPS)ANP rats (NaTc)SAP rats (NaTc)AP rats (caerulein)SAP rats neutrophils (NaTc)SAP/SIRS rats pMΦ (NaTc)	↓MDA, NF-κB, TNF-α, IL6, IL-1β, ↑SOD↑Claudin-5, Occludin↓Bip, IRE1α, TRAF2, ASK1, phosphorylation of JNK and p38 MAPK↓Bip, PERK, ATF6, IRE1↓TXB2, ↑6-keto-PGF1αHTRA1/TGF-β1 signaling pathway↑TGF-β1, EGFCa^2+^-calpain 1-caspase 12-caspase 3 signaling pathway↑ICAM-3, mCD14	[Bibr B167][Bibr B145][Bibr B141][Bibr B140][Bibr B139][Bibr B68][Bibr B29][Bibr B168][Bibr B89],b

Baicalin	*Scutellaria baicalensis* Georgi	AP rats (caerulein)	↓NF-κB, TNF-α	Xue et al., 2006
		SAP rats (NaTc)	↓TNF-α, IL-6, MDA, PLA2	Zhang et al., 2007c
		SAP rats (NaTc)	↓NO, MDA, TNF-α	Zhang et al., 2008b
		SAP rats (NaTc)	↓P-selectin, TNF-α, ↑Caspase-3	Xiping et al., 2009b
		SAP rats (NaTc)	↓IL-1β, PAF, TXB2, PLA2,↑PGE2	Zhang et al., 2009b
		SAP rats (NaTc)	↓Bcl2, ↑Bax	Xiping et al., 2009a

Resveratrol	Grapes, berries, peanuts, soya beans, red win, rhubarb, giant knotweed rhizome, *Eranthis hyemalis*	AP rats (CCK8)	↓NF-κB, TNF-α, ↑CAT, glutathione	Szabolcs et al., 2006
		AP rats (NaTc)	↓NF-κB, AP-1, TNF-α, IL-6, iNOS	Gulcubuk et al., 2014
		AP rats (caerulein)	↓IL-1β, MDA, ↑IL10, GSH-Px, SOD	Carrasco et al., 2014
		SAP rats (NaTc)SAP rats (NaTc)SAP rats (NaTc) pMΦ	↓MDA, ICAM-1, VCAM-1, TNF-α, ↑SOD↓PLA2, [Ca^2+^]i, ↑Ca^2+^-Mg^2+^-ATPase, Ca^2+^-ATPase↓NF-κB, iNOS, TNF-α, IL-1, NO	[Bibr B42][Bibr B128][Bibr B81]

Dihydro-resveratrol	Orchidaceae, *Cannabis sativa* L., A metabolite of trans-resveratrol in the human body	AP rats (caerulein)	↓MDA, NADPH oxidase, MPO, TNF-α, NF-κB, IκB degradation, AKT phosphorylation, ↑glutathione, PI3K	Tsang et al., 2016

Curcumin	Turmeric (*Curcuma longa*)	AP rats (NaTc)	↓NF-κB, AP-1	Gulcubuk et al., 2013
		SAP rats (NaTc)	↓TLR4, NF-κB	Zhong, 2015
		AP rats (NaTc)	↓TNF-α, IL6	Gulcubuk et al., 2006
		AP rats (NaTc)	↓MDA, NO, bacterial translocation	Gulcubuk et al., 2005
		AP mice (caerulein)	↑PPARγ, ↓NF-κB, TNF-α	Yu et al., 2011

Ligustrazine	*Ligusticum chuanxiong* Hort., *Curcuma aromatica* Salisb., *Jatropha podagrica* Hook	AP rats and acinar cells (caerulein)	↓p38 Erk MAPK pathways	Chen et al., 2016

Honokiol	*Magnolia officinalis Rehd.* Et Wils	SAP mice (caerulein)	↓TNF-α, IL1, NO, HMGB1, MPO	Weng et al., 2012
			↑eIF2α phosphorylated, CHOP, caspase-3	

*AP, acute pancreatitis; SAP, severe acute pancreatitis; ANP, acute necrotizing pancreatitis; SIRS, systemic inflammatory response syndrome; NaTc, sodium taurocholate hydrate; LPS, lipopolysaccharide; CCK8, cholecystokinin 8; pMΦ, peritoneal macrophages; MDA, malondialdehyde; NF-κB, nuclear factor kappa-B; TNF-α, tumor necrosis factor-α; IL, interleukin; SOD, superoxide dismutase; Bip, ER chaperone immunoglobulin-binding protein; IRE1, inositol requiring protein 1; TRAF2, TNF receptor-associated factor 2; ASK1, apoptosis signal-regulating kinase 1; JNK, c-Jun N-terminal kinase; p38 MAPK, p38 mitogen-activated protein kinases; TXB2, thromboxane-2; HTRA1, serine protease high-temperature requirement A1; TGF-β1, transforming growth factor-β1; EGF, epidermal growth factor; ICAM, intercellular adhesion molecule; mCD14, membrane-bound CD14; PLA2, phospholipase A2; NO, nitric oxide; PAF, platelet-activating factor; PGE2, prostaglandin E2; CAT, catalase; AP-1, activator protein-1; iNOS, inducible nitric oxide synthase; GSH-Px, glutathione peroxidase; MPO, myeloperoxidase; VCAM-1, vascular cell adhesion molecule 1; IκB, inhibitor protein kappa B; PI3K, phosphatidylinositol 3′-kinase; AKT, threonine kinase; TLR4, Toll-like receptor 4; HMGB1, high mobility group box 1; PPARγ, peroxisome proliferator-activated receptor gamma; CHOP, C/EBP homologous protein.*

### Emodin

**FIGURE 3:**
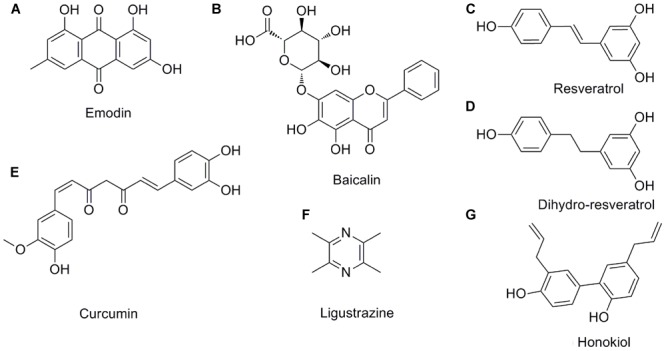
**The chemical structures of the pleiotropic natural products for AP. (A)** emodin; **(B)** baicalin; **(C)** resveratrol; **(D)** dihydro-resveratrol; **(E)** curcumin; **(F)** ligustrazine; **(G)** honokiol.

Emodin (1,3,8-trihydroxy-6-methylanthraquinone) (**Figure 3A**) is a natural anthraquinone derivative isolated from the roots and rhizomes of numerous plants, such as *Rhamnaceae, Polygonum, Liliaceae*, and *Leguminosae*, and especially, from the Chinese herb *Rheum palmatum* L., as an active compound, which has been utilized to treat critical illness (e.g., AP) in China for many years (Dong et al., 2016). Previous pharmacological studies have shown that emodin possesses biological activities such as purgative, anti-fibrotic, vasorelaxant, and immunosuppressive (Dong et al., 2016). Emodin also possesses an anti-inflammatory property in particular (Li et al., 2015). Early cell culture and animal research have revealed that emodin at the doses of 10–60 mg/kg could significantly reduce the mortality and have anti-AP effects (Wu et al., 2000, 2014; Ni et al., 2014a; Yao et al., 2015). Currently, studies suggest that emodin ameliorates AP through multiple targets; however, the exact mechanism underlying the effect of emodin in AP has not been completely addressed.

The paramount pathological manifestations of AP are pancreatic edema, hemorrhage, necrosis, and inflammatory infiltration, and increased pancreatic enzymes (Whitcomb, 2006). Emodin, as a direct NF-κB inhibitor, could result in an anti-oxidation response and subsequently suppress the expression of pro-inflammatory cytokines (Yao et al., 2015). Moreover, our previous research discovered 32 differentially expressed proteins from SAP rats with or without emodin treatment by using iTRAQ-based quantitative proteomic analysis, and a new biomarker, serine protease high-temperature requirement A1 (HTRA1), was found to associate with SAP. Further work found that one of the mechanisms underlying emodin against SAP involved inhibiting the HTRA1/TGF-β1/NF-κB signaling cascade and subsequent inflammatory responses (Li et al., 2016a).

Emodin can also promote cell apoptosis and reduce necrosis occurring in the pancreatic acinar cell or inflammatory cells, for example, emodin inhibits SIRS in SAP rats via inducing neutrophil apoptosis via the calcium-mediated caspase-12 signaling pathway (Ni et al., 2014b; Yin et al., 2016). In addition, the protective effects of emodin on ER stress responses are mediated by significantly attenuating calcium overload and decreasing the expression of ER chaperone immunoglobulin-binding protein (Bip), PERK, ATF6, IRE1α, TRAF2 and ASK1, as well as inhibiting the phosphorylation of MAPKs (Wu et al., 2013, 2014). Schmitt et al. (2004) reported that claudin and occludin expression in the pancreas are obviously reduced in AP, suggesting a possible role of tight junction disruption in interstitial edema formation. Emodin administration could increase claudin and occludin expression and reduce paracellular permeability in the pancreas (Xia et al., 2012). Furthermore, previous research indicated that emodin could reduce pancreatic ischemia and tissue injury through decreasing the expression of thromboxane-2 (TXB2) protein, which is a stable metabolite of TXA2 that has abilities to induce the deformation, release, and secretion of platelets (Kusterer et al., 1991; Wu et al., 2000). Interestingly, many studies have shown that the regeneration and repair of pancreatic injury occurs after AP (Konturek et al., 1997). Transforming growth factor-β1 (TGF-β1) is found to be involved in the pathogenesis of AP, especially pancreatic regeneration (Riesle et al., 1997). Emodin combined with somatostatin analogs may be beneficial for the up-regulation of TGF-β1 and EGF expression, which contributes to pancreatic repair and regeneration (Gong et al., 2002). Moreover, results from Konturek et al. (1998) have demonstrated that significantly up-regulated expression of epidermal growth factor (EGF) may restrict the extent of initial tissue damage and then accelerate pancreatic regeneration and repair in AP.

Emodin has a protective effect in pancreatitis-associated secondary organ damage (Wang et al., 2007; Ning et al., 2009; Xia et al., 2010; Xu et al., 2016), which is both a common serious SAP complication and a main cause of death. Pancreatitis-associated lung injury occurs as a consequence of significant pulmonary hyperemia, edema, and inflammatory infiltration in the lung tissues. Although the strategies in prevention and treatments have been improved, ALI still causes more than 50% of deaths in SAP (Bhatia, 2002). Emodin attenuates pulmonary edema and enhances alveolar epithelial barrier function by up-regulating the expression of claudin-4, claudin-5, occludin, AQP1, and AQP5 in lung tissue samples from rats with SAP-induced ALI (Xia et al., 2010; Xu et al., 2016). Several *in vivo* investigations have proposed that emodin can prevent the translocation of bacteria and endotoxins and promote the recovery of intestinal barrier function via inhibiting the intestinal mucosa cell apoptosis and up-regulating the serum leptin content (Ning et al., 2009). In addition, emodin-assisted early enteral nutrition (EAEEN) obviously abates the severity of secondary hepatic injury to function in the treatment of AP (Wang et al., 2007). However, emodin could also result in kidney toxicity, hepatotoxicity, and reproductive toxicity, particularly in long-term use with high-doses. Pharmacokinetic studies also indicated that emodin processed poor oral bioavailability because of its wide glucuronidation (Dong et al., 2016).

### Baicalin

Baicalin (5,6,7-trihydroxyflavone-7-*O*-D-glucuronic acid) (**Figure 3B**) is one of the effective flavonoid compounds extracted from the dried root of *Scutellaria baicalensis* Georgi (Zhang et al., 2009a). *In vitro* experiments of baicalin demonstrated that it features multiple activities, such as resisting bacteria, anti-inflammation, anti-oxidation, and inhibiting platelet aggregation, reducing endotoxin production and inducing apoptosis (Ueda et al., 2002; Shen et al., 2003). The pharmacological actions of baicalin are quite similar to those of octreotide, which is a somatostatin analog that can significantly inhibit pancreatic secretion (Shen et al., 2003; Tian et al., 2009). Fortunately, animal researches indicated that baicalin (50–100 mg/kg) has diverse pharmacological actions associated with antagonizing many stages of SAP onset and is much cheaper than octreotide, thus implying that baicalin is a promising medication for AP treatment. Early studies reported that baicalin could inhibit digestive enzyme activity, reduce pancreas necrosis, restrict lipid peroxidation, and increase the SAP rat survival rate (Zhang et al., 2007c, 2009a).

The beneficial effects of baicalin appear to be mediated by inhibiting NF-κB and TNF-α activity and increasing caspase-3 expression in multiple organs (Xue et al., 2006). Furthermore, baicalin may be applied for decreasing the expression levels of the P-selectin protein, which serves as a marker for leukocyte-endothelial cell adhesion and active leukocyte-mediated organ injury and plays a key role in the progression of AP (Xiping et al., 2009b). Complementarily, early treatment with baicalin exerts significant protective effects on SAP-induced multiple organ injuries, such as liver (Zhang et al., 2008a), kidney (Zhang et al., 2007b), intestinal mucosa (Zhang et al., 2009a), heart (Xiping et al., 2007), and thymus (Xiping et al., 2010), with their possible mechanisms associated with inhibiting inflammatory mediators and inducing apoptosis.

### Resveratrol

Resveratrol (trans-3,5,4′-trihydroxystilbene) (**Figure 3C**), as a naturally polyphenolic phytoalexin, exists in almost 70 vegetables and fruits or food products, especially in grapes and red wine. It also has been identified as a major active ingredient in giant knotweed rhizome and rhubarb in TCM (Ma et al., 2011). Resveratrol is considered to be a good candidate for the treatment of oxidative and/or inflammatory diseases due to its antioxidant and anti-inflammatory activities and ease of extraction (Ma et al., 2011). Moreover, it has received an increasing amount of attention in treating AP (Meng et al., 2005b; Szabolcs et al., 2006). Previous animal studies have identified and documented the role of resveratrol (10–30 mg/kg) in decreasing the levels of amylase and lipase enzymes, mitigating histological damage in the pancreas and extra-pancreatic organs and reducing the mortality in the case of AP, indicating the therapeutic effect of resveratrol against AP (Li et al., 2006; Wang et al., 2008).

Resveratrol can decrease the production and release of pro-inflammatory mediators, such as IL-1, IL-6, IL-8, TNF-α, and NO, via blocking the activation of NF-κB and AP-1 and the associated kinases and increasing anti-inflammatory cytokine IL-10 levels (Ma et al., 2005; Szabolcs et al., 2006; Carrasco et al., 2014; Gulcubuk et al., 2014). At the same time, resveratrol suppressed the expression of intercellular adhesion molecule-1 (ICAM-1), which could mediate leukocyte adhesion to endothelium and reduce the infiltration of leukocytes into inflammatory sites (Meng et al., 2005b; Ma et al., 2011; Jha et al., 2012).

Leonard et al. (2003) demonstrated that resveratrol can clear hydroxyl superoxides and metal inductive radicals in AP as a high-efficiency scavenger. The results from recent studies also demonstrated that resveratrol exerts a protective effect against lipid peroxidation in the membrane and prevents DNA damage via the inhibition of the NF-κB pathway and subsequent suppression of ROS products (Meng et al., 2005a; Li et al., 2006; Ma et al., 2011). In addition, it has been observed that SOD activity decreases, and the MDA activity significantly increases in organs in the early phases of AP; resveratrol reverses these phenomena to reduce the AP induced oxidative damage in the pancreas (Li et al., 2006).

Another advantage of resveratrol is that it also appears to reduce the intracellular calcium overload through restoring the intracellular calcium regulatory mechanisms, which limit not only pancreatic tissue injury but also secondary organ injury (Wang et al., 2008). Studies of SAP have shown that resveratrol may stabilize erythrocytes, improve the decrease of blood flow, decrease blood viscosity and leukocyte-endothelial interaction, thus decreasing thrombus formation and ameliorating the hypercoagulable state existing in every stage of SAP (Sha et al., 2013). Moreover, resveratrol could suppress microcirculatory disturbance via inhibition of the renin-angiotensin system (RAS) system and regulation of an unbalanced ET/NO status (Ma et al., 2011).

Resveratrol also has the ability to relieve injury in extra-pancreatic organs in SAP such as intestines, liver, brain and lungs through the up-regulation of Bcl-2 and the down-regulation of Bax, cytochrome *c*, and caspase-3 levels (Meng et al., 2005b; Jha et al., 2008, 2009; Sha et al., 2008). In short, resveratrol has shown potential therapeutic effects in cases of AP by inhibiting the release of inflammatory mediators, promoting antioxidant effects, regulating cytoplasm calcium homeostasis, reversing microcirculatory disturbance, and inducing apoptosis.

When orally consumed, *trans*-resveratrol is rapidly metabolized in the human colon by gut bacteria and converted to dihydro-resveratrol (3,5,4′-trihydroxy bibenzyl), **Figure 3D** (Tsang et al., 2016). Importantly, the solubility of dihydro-resveratrol was at least five times higher than *trans*-resveratrol while exhibiting a much lower cytotoxicity; thus, dihydro-resveratrol is particularly suitable for patients unresponsive to *trans*-resveratrol due to the lack of proper microbial strains (Tsang et al., 2016). Lin et al. (2016) and Tsang et al. (2016) demonstrated that dihydro-resveratrol could significantly ameliorate pancreatic oxidative damage and AP-associated lung injury. The underlying molecular mechanisms involve in the decreased production of intracellular reactive oxidative products and pro-inflammatory cytokines, and the inhibition of the NF-κB and phosphatidylinositol 3′-kinase (PI3K)-serine/threonine kinase (AKT) signaling pathways.

### Curcumin

Curcumin (diferuloylmethane, **Figure 3E**) is a turmeric polyphenol derived from *Curcuma longa* (turmeric), which has been prized in Ayurvedic medicine since ancient times for the treatment of inflammatory conditions due to its various pharmacological benefits including antioxidant and anti-inflammatory properties (Jurenka, 2009; Shehzad et al., 2013). Based on the results of cell cultures, animal models and clinical trials, ample evidence validates that curcumin at the doses of 100 mg/kg may have potential as a therapeutic agent in AP (Gulcubuk et al., 2013; Zhong, 2015). Curcumin’s improvement of AP is achieved through repressing the infiltration of inflammatory cells, inhibiting lipid peroxidation, and regulating TLR-4/NF-κB and PPARγ/NF-κB signaling pathway, preventing free radical injury and the prevalence of bacterial translocation, thereby reducing trypsin activation, oxidative enzymes, and tissue injury (Gulcubuk et al., 2005, 2006, 2013; Yu et al., 2011; Zhong, 2015). Moreover, curcumin suggests that the activation of caspase-3 may lead to an increase in apoptosis in the early and late phases of experimental AP.

### Others

Ligustrazine (tetramethylpyrazine, **Figure 3F**) (Chen et al., 2016) and honokiol (3,5′-diallyl-4,2′-dihydroxybiphenyl, **Figure 3G**) (Weng et al., 2012) possess special pharmacological activities associated with the acceleration of acinar cell apoptosis at an early phase of AP. Thus, they may soon join the list of candidate drugs in the treatment of AP besides the above-mentioned natural products. However, in-depth research is necessary to confirm this hypothesis.

## Network Target Prediction

Traditional Chinese medicine is one of the promising strategies in treating AP due to its multi-targeting and lower side effects (Yao et al., 2016a). Despite massive investments in TCM research and development, there has been no significant increase in the number of new drugs approved or translated for clinical use. Single targeted drug discovery has proved to be ineffective in combating complex diseases that harbor robust biological networks such as AP. Network pharmacology prediction provides a highly useful method for drug targets, which differs from conventional single target intervention by integrating network biology and polypharmacology, thereby exploring drugs to target numerous proteins or networks involved in a disease (Cheng et al., 2015; Gao et al., 2016). In this review, we also discuss the application of network pharmacology for AP innovative drugs discovery.

**FIGURE 4:**
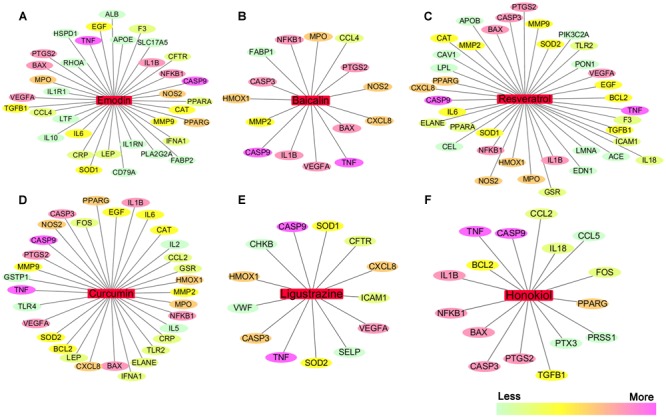
**The natural product-target network predicted by network pharmacology. (A–F)** In this network, red rectangles correspond to natural products (emodin, baicalin, resveratrol, curcumin, ligustrazine, and honokiol), and different colors-ellipses correspond to targets. The colors of each target transit from green to purple, indicating its increased importance in the process of these natural products for AP treatment. In the relationships between natural products and targets, resveratrol corresponds to the highest number of candidate targets (degree = 38), followed by emodin (degree = 35), curcumin (degree = 32), honokiol (degree = 16), baicalin (degree = 15), and ligustrazine (degree = 13). The candidate targets in the progression of AP mainly related to inflammation (TNF, NFKB1, PTGS2, IL1B, IL6, IL10, etc.), apoptosis (CASP9, CASP3, BAX, BCL2, etc.), oxidation–reduction (NOS2, SOD1, MPO, CAT, etc.), and lipid metabolic process (PPARA, PPARG, APOE, APOB, etc.), as well as the maintenance of Ca^2+^ homeostasis (ELANE, CAV1, CCL4, etc.) and microcirculation (IFNA1, SELP, etc.).

We searched the TCMID, DrugBank, GeneCards, and STITCH databases to validate 68 AP targets based on six pure natural products (emodin, baicalin, resveratrol, curcumin, ligustrazine, and honokiol) mentioned above. Subsequently, a compound-target network was constructed using Cytoscape 3.3.0 software (Cheng et al., 2015; Xiang et al., 2016). As shown in **Figures 4A–F**, in this network, red rectangles and different colors-ellipses, respectively, correspond to natural products and targets, and the colors of each target transit from green to purple, indicating its increased importance in the process of these natural products for AP treatment. In the relationships between natural products and targets, resveratrol corresponds to the highest number of candidate targets (degree = 38), followed by emodin (degree = 35), curcumin (degree = 32), honokiol (degree = 16), baicalin (degree = 15), and ligustrazine (degree = 13).

These emphasize the integral function of natural products in the treatment of AP through providing multiple therapeutic effects on multiple targets as compared with Western medicine, which usually focuses on a single target. The candidate targets in the progression of AP mainly related to inflammation (TNF, NFKB1, PTGS2, IL1B, IL6, IL10, etc.), apoptosis (CASP9, CASP3, BAX, BCL2, etc.), oxidation–reduction (NOS2, SOD1, MPO, CAT, etc.), and lipid metabolic process (PPARA, PPARG, APOE, APOB, etc.), as well as the maintenance of Ca^2+^ homeostasis (ELANE, CAV1, CCL4, etc.) and microcirculation (IFNA1, SELP, etc.). Especially, inflammation and apoptosis-related targets (e.g., TNF, CASP9, NFKB1, BAX, IL1B, CASP3) show the higher correlation (degree = 6 or 5), demonstrating the potential therapeutic effect of these natural products on AP through modulating these relevant proteins. Except for the common targets, each natural product has specific regulation on some targets. For instance, resveratrol has a potential regulatory effect on CAV1 that promotes the maintenance of intracellular calcium homeostasis; emodin maybe benefit to regulate blood lipid levels in AP by regulating the expression and function of APOE that contributes to the lipoprotein biosynthetic, catabolic, and metabolic process; ligustrazine may improve the state of hypercoagulability and microcirculation during AP via regulating VWF and SELP expression. Each natural product acts on multiple targets, and the different natural products share the synergistic targets, which is the basis of action of pure natural products. However, since the compound-target network is only obtained by data mining combined with computer simulation predictions, whether the above natural products against AP through these potential targets still require further experimental validation.

## Potential Toxic Natural Products for Pancreas

Recent research pointed out an increasing interest concerning the health benefits of natural products, and they have been considered a good complementary and alternative medicine in the treatment of AP. As a coin has two sides, exaggerating the safety and non-toxic advantages of natural products leads to the negligence of potential pancreas to toxicity for a long time. There are very limited data of about the potential toxicity of herbs or natural products on the pancreas. Currently, three case reports showed a probable AP induced by saw-palmetto, a phytotherapeutic agent for symptoms related to benign prostatic hyperplasia (BPH). It has been postulated that saw-palmetto stimulates estrogenic receptors and then increases triglyceride levels or induces a hypercoagulable state that leads to pancreatic necrosis (Jibrin et al., 2006; Wargo et al., 2010; Bruminhent et al., 2011). The evidence derived mainly from random case reports described possible *Ceramium kondoi* or horsetail infusions induced AP (Kim et al., 2013; Garcia Gavilan et al., 2017). Experimental results indicate that l-cyano-2-hydroxy-3-butene (CHB), a nitrile derived from many cruciferous plants, is a selective pancreatotoxin. CHB is also a possible inducer of tissue glutathione in the pancreas, even at toxic doses (200 mg CHB/kg body weight) (Wallig et al., 1988). L-canavanine extracting from *Hedysarum alpinum* seeds processes potential pancreatotoxicity when it’s severs as an antitumor. Histological researches of tissues from rats treated with canavanine (3.0 g/kg) for 6 days revealed pancreatic acinar cell atrophy and fibrosis; and serum amylase and lipase levels were increased after one sc injection of 2.0 g/kg canavanine (Thomas and Rosenthal, 1987). Overall, natural products have the potential to be not only beneficial but also harmful under a number of medical conditions. This information should prompt clinicians to consider natural product a potential cause of AP.

## Conclusion

**FIGURE 5:**
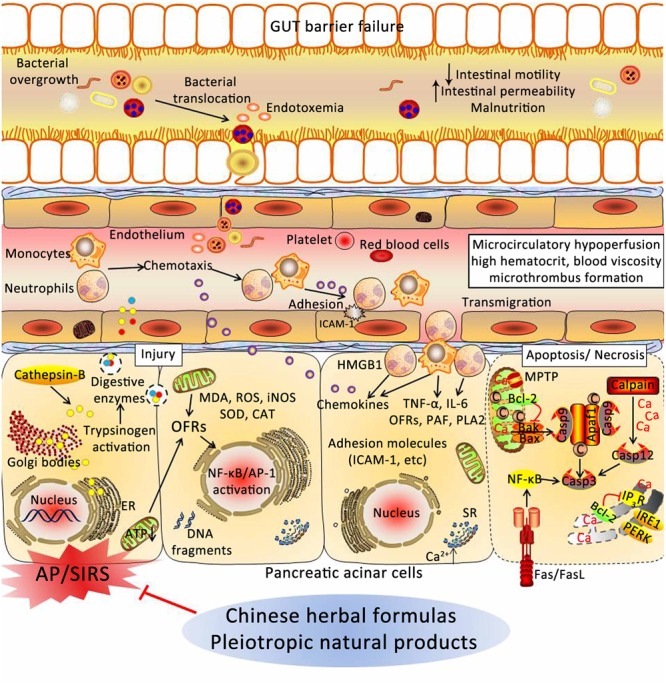
**Underlying mechanisms of Chinese herbal medicines (CHMs) for the treatment of AP.** CHMs may prevent cellular damage in pancreas associated with AP through a variety of mechanisms, including: (1) depressing the synthesis and secretion of digestive enzymes; (2) depressing oxidative stress through increased antioxidant levels and decreased the excessive OFRs; (3) inhibiting activation of inflammatory pathways; (4) relieving ER stress via PERK and IRE1; (5) restoring the intracellular calcium regulatory mechanisms; (6) inducing the switch from apoptosis to necrosis in pancreatic cells; (7) increasing pancreatic blood flow, and reducing blood viscosity; and (8) restoring intestinal barrier function and blocking bacterial translocation. AP, acute pancreatitis; SIRS, systemic inflammatory response syndrome; ER, endoplasmic reticulum; ATP, adenosine triphosphate; OFRs, oxygen free radicals; MDA, malondialdehyde; ROS, reactive oxygen species; iNOS, inducible nitric oxide synthase; SOD, superoxide dismutase; CAT, catalase; NF-κB, nuclear factor kappa-B; AP-1, activator protein-1; HMGB1, high mobility group box 1; TNF-α, tumor necrosis factor-α; IL-6, interleukin-6; PAF, platelet-activating factor; PLA2, phospholipase A2; ICAM-1, intercellular adhesion molecule-1; SR, sarcoplasmic reticulum; MPTP, mitochondrial permeability transition pore; IP3R, inositol 1,4,5-trisphosphate receptor; PERK, RNA-activated protein kinase-like ER kinase; IRE1, inositol requiring protein 1; Apaf-1, apoptotic protease activating factor-1.

Acute pancreatitis is the leading cause of hospital admission for gastrointestinal diseases, and the effective strategies are limited. Thus, it is urgent to search ways to prevent and treat AP. In this review, experimental evidence implicates CHMs in the prevention and treatment of AP through various underlying mechanisms, including: (1) depressing the synthesis and secretion of digestive enzymes; (2) depressing oxidative stress through increased antioxidant levels and decreased the excessive OFRs; (3) inhibiting activation of inflammatory pathways; (4) relieving ER stress via PERK and IRE1; (5) restoring the intracellular calcium regulatory mechanisms; (6) inducing the switch from apoptosis to necrosis in pancreatic cells; (7) increasing pancreatic blood flow, and reducing blood viscosity; and (8) restoring intestinal barrier function and blocking bacterial translocation (**Figure 5**). However, more convincing studies are needed to confirm this hypothesis.

## Author Contributions

HX, QZ, BQ, XT, SX, HS, JQ, and DS wrote the manuscript.

## Conflict of Interest Statement

The authors declare that the research was conducted in the absence of any commercial or financial relationships that could be construed as a potential conflict of interest.
